# Integrating Casemix system in to the Philippine social health insurance

**DOI:** 10.1186/1472-6963-12-S1-I6

**Published:** 2012-11-21

**Authors:** Maria Josefina A Gool

**Affiliations:** 1 Philippine Health Insurance Corporation, Philippines

## 

The Philippine Health Insurance Corporation (PhilHealth) is a government owned and controlled corporation, established in 1995 and tasked to implement the National Health Insurance Program. Since its inception, PhilHealth has utilized the fee-for-service scheme for majority of inpatient benefits and case payment for selected, mostly outpatient benefits.

In 2010, the country elected a new president and Universal Health Care was declared a national agenda. The challenge for PhilHealth was not only to ensure 100% coverage but provide true financial risk protection. One of the identified key strategies was to shift to a new provider payment mechanism that will foster patient empowerment through clear and predictable financial support. In Sept 2011, PhilHealth rolled out the first 23 medical and surgical case rates which accounts for 45% of all cases being reimbursed by PhilHealth the previous years. Rates were determined by assigning weights to previously obtained tariff rates, contracting rates for public and private hospitals and average value per claim for the preceding years. The highest computed rates were identified and used. Along with this, a zero co-payment policy or No Balance Billing policy was instituted.

After almost 8 months of implementation, case payment scheme is proving to be the only solution for PhilHealth to ensure member empowerment, cost containment and claims processing efficiency. However, the problem still lies in government not being able to provide the necessary medicines and services that is already included in the case payment package. Despite these challenges, 29 medical and 32 surgical illnesses more will be paid as case rates. This will then account for 85% of cases being reimbursed by PhilHealth until a time that these case rates will be converted to full case mix.

The change in payment provider mechanism entails a big paradigm shift for the whole health system. Reform interventions inside the corporation are being done that will truly inform, empower and guide its members. Expansion of benefit packages has started. Information technology infrastructure is in full swing and new engagement processes with providers are being adapted. In the end, universal health care can be achieved by re-awakening the spirit of solidarity among Filipinos, which is the very essence of the National Health Insurance Program.

